# Monoclonal antibody-based capture ELISA in the diagnosis of previous dengue infection

**DOI:** 10.1186/s12985-019-1222-9

**Published:** 2019-10-29

**Authors:** Chukiat Sirivichayakul, Kriengsak Limkittikul, Pornthep Chanthavanich, Sutee Yoksan, Anuttarasakdi Ratchatatat, Jacqueline Kyungah Lim, Watcharee Arunsodsai, Arunee Sabchareon

**Affiliations:** 1 Department of Tropical Pediatrics, Faculty of Tropical Medicine, Mahidol University, Bangkok, Thailand; 2 Center for Vaccine Development, Mahidol University, Nakhonpathom, Thailand; 3 Department of Disease Control, Ministry of Public Health, Nonthaburi, Thailand; 4 Global Dengue and Aedes-Transmitted Diseases Consortium (formerly Pediatric Dengue Vaccine Initiative), International Vaccine Institute, Seoul, South Korea

**Keywords:** Dengue, Plaque reduction neutralization test, Enzyme-linked immunosorbent assay, Monoclonal antibody

## Abstract

**Background:**

Dengue is an important mosquito-borne disease. There is currently only one licensed vaccine for dengue prevention. The vaccine provides higher efficacy in pre-vaccination dengue-seropositive persons but a higher risk of subsequent more severe dengue in dengue-seronegative persons. It is recommended that the dengue vaccine may be given in dengue-seropositive individuals or as mass vaccination without individual pre-vaccination screening in areas where the dengue seroprevalence is > 80% in children aged 9 years. We evaluated a dengue specific immunoglobulin G monoclonal antibody-based capture enzyme-linked immunosorbent assay (MAb-ELISA) in the diagnosis of previous dengue infection using serum samples from the cohort study in Ratchaburi Province, Thailand.

**Methods:**

The MAb-ELISA was compared to 70% plaque reduction neutralization test (PRNT70) in 453 serum samples from children aged 3–11 years in Ratchaburi Province, Thailand.

**Results:**

The sensitivity and specificity of MAb-ELISA at the positive to negative (P/N) ratio cut-off level of > 3 were both 0.91 in the diagnosis of previous dengue infection, compared to PRNT70. The false positivity was mainly in Japanese encephalitis (JE) seropositive subjects.

**Conclusions:**

This research provides evidence that MAb-ELISA is useful for dengue seroprevalence study and dengue pre-vaccination screening. JE seropositivity was the major cause of false positive result in the study population.

## Background

Dengue is an important mosquito-borne disease in the tropics with rapidly increasing incidence and expanding endemic areas. There has been no specific treatment for dengue but currently, one dengue vaccine is licensed. This tetravalent chimeric yellow fever-dengue vaccine (Dengvaxia®) has been approved for the prevention of dengue in children and adults aged 9–45 years. In its phase 2b and phase 3 trials, the overall protective efficacy ranged from 30.2 to 60.8% [1–3]. Dengue vaccination may have high cost-effectiveness and public health impact in areas with high dengue seroprevalent rate, particularly if the rate is > 70% [4, 5]. The vaccine provided higher efficacy in pre-vaccination dengue-seropositive persons but a higher risk of subsequent more severe dengue in pre-vaccination dengue-seronegative persons [6, 7]. The World Health Organization Strategic Advisory Group of Experts on immunization (SAGE) recommends that dengue vaccination in only dengue-seropositive persons is the preferred option and pre-vaccination screening test should be performed using highest specific tests to minimize the inadvertent use of the vaccine in seronegative persons [8]. Mass vaccination without individual pre-vaccination screening may also be considered in areas where the dengue seroprevalence is > 80% in children aged 9 years [9]. A highly specific and sensitive test for dengue serostatus is essential for both approaches.

Among various dengue antibody tests, the plaque reduction neutralization test (PRNT) is accepted as the gold standard. It assesses antibodies that neutralize and prevent virions from infecting cultured cells and is currently the most virus-specific serological test among the flaviviruses and serotype-specific test among the dengue viruses [10]. Other tests that can be used in assessing the existence of dengue antibody include dengue NS1 antibody enzyme-linked immunosorbent assay (ELISA) [11], dengue-specific antibody ELISA [12] and hemagglutination inhibition test. These antibody tests, however, may be inaccurate in assessing dengue serostatus due to the waning of antibody causing false negativity, or cross-reactive antibody with other flavivirus causing false positivity. To the best of our knowledge, there has been no study primarily aiming to evaluate the accuracy of the dengue specific immunoglobulin G (IgG) monoclonal antibody-based capture enzyme-linked immunosorbent assay (MAb-ELISA) in the assessment of dengue serostatus.

The objective of this report was to evaluate the sensitivity and specificity of MAb-ELISA compared to 70% plaque reduction neutralization test (PRNT70) for the assessment of dengue serostatus.

## Methods

This was a retrospective study nested in a prospective study of the epidemiology of dengue in a cohort of 3015 primary school children aged 3–11 years at enrolment in Ratchaburi Province, Thailand conducted from 2006 to 2009 [13]. In the major cohort study, we prospectively collected baseline serum samples from all subjects in 2006. The MAb-ELISA was tested in all blood samples and PRNT70 was randomly tested in a subset of approximately 15% of these 3015 blood samples (*N* = 453). This report describes the laboratory data from this subset. The results from the MAb-ELISA was compared to the results of PRNT70. In order to compare the performance of the two tests, a receiver operating characteristic (ROC) curve was constructed and an appropriate cut-off level was identified with optimal sensitivity and specificity for the diagnosis of previous dengue infection.

The proportion of 15% from approximately 3000 subjects was considered to be adequate to test a hypothesis of at least 5% difference between PRNT70 and MAb-ELISA with confidence level 0.97 and expected seropositive rate 50%.

All blood samples were drawn into serum separator tubes, allowed to clot at room temperature for 1–2 h, then stored at 4 °C. Sera were separated into aliquots within 24 h and stored at -70 °C until laboratory testing. All tests were performed at the Center for Vaccine Development, Institute of Molecular Biosciences, Mahidol University, Salaya, Nakhonpathom, Thailand.

For PRNT70, the method was modified from that described by Russell et al. [14]. All four dengue serotypes were tested. Monkey kidney-derived LLC-MK2 cells were used for virus production and PRNT. The dengue viruses (D) used in the assay were D1 (16007), D2 (16681), D3 (16562), and D4 (1036). As Thailand is an endemic area of Japanese encephalitis (JE), antibody to JE virus (Beijing strain) was also included in the assay. LLC-MK2 cells were seeded in 6-well plates at 1 × 10^5^ cells/well, and incubated for 6–8 days. The serum samples were diluted for a single dilution of 1:30 using phosphate buffer solution (PBS) pH 7.5 with 30% fetal bovine serum, mixed with the virus and incubated. Following infection, cells were overlaid with 3.0% carboxymethyl cellulose with neutral red added. Plaques were visualized and counted after cultivation for 7 days. The serum samples that reduced the number of plaques by 70% of any dengue serotype compared to negative control were considered positive.

For MAb-ELISA, the test was slightly modified from that described previously [15]. Briefly, the ELISA plates were prepared by dispensing 75 μl of diluted purified dengue monoclonal antibody (2H2) in carbonate buffer pH 9.0 into each well and incubated 24 h at 4^o^ C and washed with PBS-Tween 20 (PBST). Then, the plates were blocked with 150 μl of 5% non-fat dried milk in PBS (NDM-PBS) for 1 h at 37^o^ C and washed again with PBST and dispensed with the dengue antigens. The dengue antigens were prepared by diluting Vero cultured mixed D1, 2, 3, and 4 to 1:4 in 5% NDM-PBS. Seventy-five μl of diluted dengue antigens was dispensed into the plates in the adjacent alternative wells. The plates were then incubated 1 h at 37^o^ C and washed. All serum controls and serum samples were diluted to 1:400 with the ELISA diluents and 75 μl of diluted controls and serum samples were placed into duplicated well and incubated 1 h at 37^o^ C and washed. Then, 75 μl of diluted horseradish peroxidase-conjugated anti-human IgG was added into each of the test wells. The plates were incubated 1 h at 37^o^ C and washed. After washing, 100 μl of 4 mg o-phenylene diamine (Dako, Denmark) in 10 ml citrate phosphate buffer and 33 μl fresh 3% H_2_O_2_ were added to each of the test wells and the plates were incubated at room temperature in the dark for 15 min. The reaction was then stopped by adding 50 μl of 4 M H_2_O_2_ into each well. The Absorbance was read at wavelength 492 nm. The results were expressed as positive to negative (P/N) ratio where the positive value was the optical density (OD) of serum samples and the negative value was the OD of normal negative control serum.

## Results

Among 3015 students aged 3–13 years enrolled in the major study, both PRNT70 and MAb-ELISA were performed in serum samples of 453 (15%) subjects and laboratory data from these 453 subjects were used for this particular analysis.

**Fig. 1:**
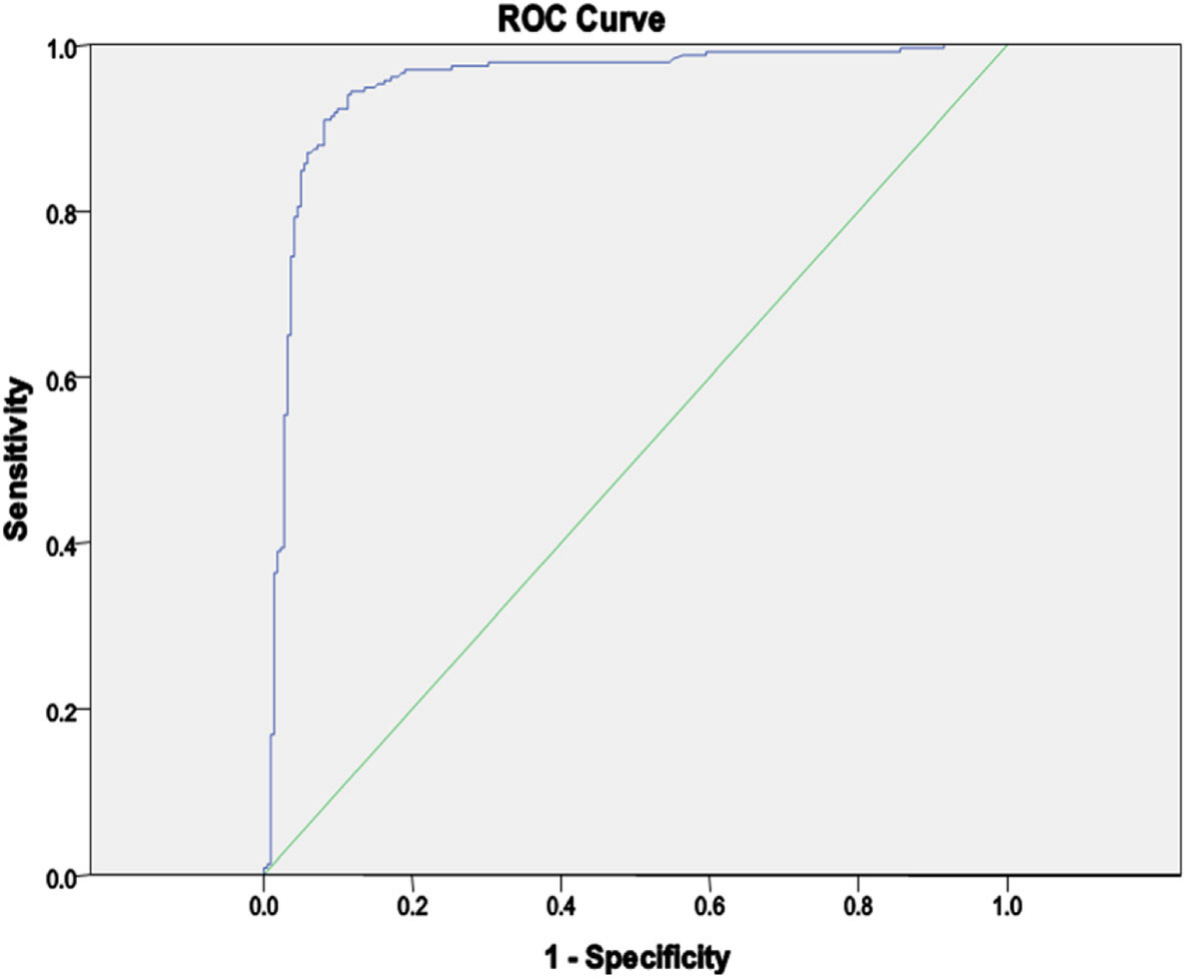
The receiver operating characteristic curve of MAb-ELISA compared to PRNT70. The green (lower) line represents the diagonal reference and the blue (upper) line indicates the performance of MAb-ELISA. With the blue line close to the top-left corner, the ROC curve supports that the MAb-ELISA is highly accurate, consistent with the results of the PRNT70

It was found that the P/N ratio in MAb-ELISA ranged from 0.11 to 58.63. The ROC curve (Fig. 1) reveals a high sensitivity with high specificity when the diagnostic performance of MAb-ELISA was compared against PRNT70. The green (lower) line represents the diagonal reference line which shows no predictive value along the diagonal, and the blue (upper) line indicates the actual test. Figure 1 reveals that the blue line is close to the top-left corner. The area under this curve is 0.95 and standard error 0.11. These data indicate that the MAb-ELISA is highly accurate, consistent with the results of the PRNT70.

Table 1 shows a comparison between PRNT70 and MAb-ELISA at different cut-off levels. Most of the PRNT70 confirmed dengue seropositive cases had MAb-ELISA P/N ratio > 5 and most of the PRNT70 confirmed dengue seronegative cases had MAb-ELISA P/N ratio < 3. Table 2 shows the sensitivity, specificity, as well as positive predictive value (PPV) and negative predictive value (NPV) of MAb-ELISA compared to PRNT70 at different cut-off levels. The P/N ratio cut-off level of > 3 shows optimal sensitivity and specificity estimates with more than 90% for both PPV and NPV. At this cut-off level and considering PRNT70 as the gold standard, the sensitivity and specificity of MAb-ELISA in the diagnosis of previous dengue infection were both 0.91. JE seropositivity was the major cause of false positive MAb-ELISA (14 out of 20 children).

**Table 1:** Comparison between PRNT70 and MAb-ELISA in detecting exposure to dengue

MAb-ELISA (P/N ratio)	PRNT70					
	Dengue positive			Dengue negative		
	JE positive	JE negative	Total	JE positive	JE negative	Total
> 5	121	75	196	7	4	11
4 to < 5	4	4	8	6	1	7
3 to < 4	4	3	7	1	1	2
2 to < 3	5	2	7	6	2	8
1.5 to 2	3	3	6	10	4	14
1 to < 1.5	2	0	2	21	22	42
< 1	2	3	5	62	75	137
Total	141	90	231	113	109	221

**Table 2:** Sensitivity, specificity, positive predictive value and negative predictive value of MAb-ELISA at different cut-off levels compared to PRNT70

MAb-ELISA (P/N ratio)	Sensitivity (95% C.I)	Specificity (95% C.I)	Positive predictive value (95% C.I)	Negative predictive value (95% C.I)
> 1	97.84 (95.02–99.29%)	61.99 (55.24–68.42%)	72.90 (69.43–76.12%)	96.48 (91.96–98.50%)
> 1.5	96.97 (93.86–98.77%)	81.00 (75.19–85.95%)	84.21 (80.23–87.51%)	96.24 (92.48–98.15%)
> 2	94.37 (90.57–96.97%)	87.33 (82.21–91.41%)	88.62 (84.61–91.68%)	93.69 (89.73–96.19%)
> 3	91.34 (86.95–94.63%)	90.95 (86.37–94.38%)	91.34 (87.39–94.14%)	90.95 (86.84–93.87%)
> 4	88.31 (83.45–92.15%)	91.86 (87.43–95.10%)	91.89 (87.89–94.65%)	88.26 (84.03–91.48%)
> 5	84.85 (79.56–89.21%)	95.02 (91.27–97.49%)	94.69 (90.90–96.95%)	85.71 (81.53–89.07%)

## Discussion

The ideal laboratory test for dengue serostatus should be highly sensitive and specific, cheap, easy to perform, and reproducible. Hemagglutination inhibition test is easy and cheap but has low specificity. PRNT is highly specific but is time consuming, laborious, expensive, and still need standardization [10]. ELISA is closest to the definition of the ideal test.

Although there have been many available in-house and commercial immunoassays that detect dengue-specific antibody, almost all of these tests were primarily used to diagnose acute dengue infection. Nevertheless, a study on the evaluation of six immunoassays for detection of dengue IgG revealed sensitivity ranged from 0.52 to 1.0 and specificity ranged from 0.86 to 1.0 [16]. The only one ELISA test that was specifically developed to evaluate the dengue exposure is anti-dengue NS1 IgG ELISA [11]. This assay was also shown to be highly sensitive and specific with the ROC area under the curve > 0.9, similar to our MAb-ELISA.

The principle of MAb ELISA is utilizing dengue-specific monoclonal antibody to coat onto the ELISA plate. This monoclonal antibody will capture the dengue antigens onto the plate. It is more practical than directly coating the plate with purified dengue virus (indirect ELISA) and is more sensitive and more specific than coating the plate with anti-human IgG (antibody-capture ELISA) [16].

In this study, we used a single dilution PRNT70 as a gold standard because this method is qualitative but is more convenient, cheaper and less laborious compared to standard PRNT50. We had data on PRNT50 in baseline serum samples in 8 children. The comparison between PRNT70 and PRNT50 in these 8 children showed 100% consistency between both tests, i.e. 7 cases were dengue seropositive and one case was dengue seronegative (data not shown).

This study confirms that MAb-ELISA has high sensitivity and specificity in diagnosing previous dengue infection. The P/N ratio cut-off level of 3 is more suitable because of the optimal sensitivity and specificity (0.91 for both sensitivity and specificity compared to PRNT70). This test is not expensive, can be tested in high amount and therefore is more suitable for dengue seroprevalence study and pre-vaccination screening. The false positivity and false negativity in this MAb-ELISA may be due to the waning of dengue antibody to an undetectable level in baseline serum samples or previous JE infection/vaccination induced cross-reactive antibody response. When using this test in dengue seroprevalence study, it is reasonable to estimate that the real seroprevalence may be slightly higher than the rate detected by MAb-ELISA. When this test is used for dengue pre-vaccination screening, it should be carefully explained to both seropositive and seronegative individuals on the limitation of the test, i.e. its positive and negative predictive values. The chance of true positive MAb-ELISA may be lower in areas where the incidence of dengue is lower or the incidence of JE infection/vaccination is high. We have no data on the cross-reactive antibody from other flavivirus infections on this MAb-ELISA. However, based on a previous study that the detection of flavivirus specific IgG using either an immunofluorescence assay or an enzyme immunoassay showed high cross-reactions with other flavivirus infections [17], we believe that this test should also have some cross-reactivity. Moreover, a recently emerged Zika virus is closely related to dengue virus [18] and the antibody to Zika virus exhibited cross-reactivity to dengue antigens [19]. Although this study was conducted in the serum samples collected prior to the first demonstration of Zika virus circulation in Thailand [20], it is still possible that Zika virus might be endemic in the study area and caused the false dengue seropositivity.

## Conclusion

MAb-ELISA is highly sensitive and specific, compared to PRNT70, for the assessment of dengue serostatus and is useful for dengue seroprevalence study and dengue pre-vaccination screening. The P/N ratio cut-off level of > 3 provides optimal sensitivity and specificity (both 0.91). JE seropositivity was the major cause of false positive result in the study population.

## Data Availability

The datasets used and/or analyzed during the current study are available from the corresponding author on reasonable request.

## Abbreviations

ELISA: Enzyme-linked immunosorbent assay
IgG: Immunoglobulin G
JE: Japanese encephalitis
MAb-ELISA: Dengue specific immunoglobulin G monoclonal antibody-based capture enzyme-linked immunosorbent assay
NDM-PBS: Non-fat dried milk in PBS
NPV: Negative predictive value
OD: Optical density
P/N ratio: Positive to negative ratio
PBS: Phosphate buffer solution
PBST: PBS-Tween 20
PPV: Positive predictive value
PRNT: Plaque reduction neutralization test
PRNT70: 70% plaque reduction neutralization test
SAGE: Strategic Advisory Group of Experts on immunization
